# Communicating Hydrocephalus After Radiotherapy for a Vestibular Schwannoma

**DOI:** 10.7759/cureus.91903

**Published:** 2025-09-09

**Authors:** Htet Htet Myo Set, Khin Ni Sann, Ramkumar Shanmugasundaram

**Affiliations:** 1 Cancer Center, University Hospital Southampton NHS Foundation Trust, Southampton, GBR

**Keywords:** communicating, hydrocephalus, non-obstructive, radiotherapy, vestibular schwannoma

## Abstract

Hydrocephalus is a known tumour complication associated with vestibular schwannomas, which can be either the obstructive or communicating (non-obstructive) type. The latter represents a rare and controversial complication following radiotherapy, typically resulting from tumour necrosis. Management generally involves cerebrospinal fluid diversion. Here, we present a case of normal-pressure and communicating hydrocephalus that developed after radiotherapy for a vestibular schwannoma. The patient was a 72-year-old woman who initially presented with imbalance and right sensorineural hearing loss. An MRI scan identified an enhancing mass in the right cerebellopontine angle and the right internal auditory meatus, consistent with a diagnosis of a right vestibular schwannoma. Following evidence of increased tumour growth on subsequent MRI scans, the patient underwent radiotherapy with a total dose of 50 Gy delivered in 30 fractions, utilising the volumetric modulated arc therapy technique. Sixteen months after radiotherapy, the patient experienced new neurological symptoms, including intermittent slurred speech, loss of concentration, slow cognition, right facial asymmetry, numbness, and recurrent falls. CT of the head showed communicating hydrocephalus. Therefore, a left ventriculoperitoneal shunt was subsequently inserted for the management of hydrocephalus. However, due to shunt blockage, the patient underwent near-total resection of the enlarging vestibular schwannoma and has since been followed by the neurosurgical team.

## Introduction

Vestibular schwannomas are benign tumours of the vestibular nerve, typically presenting with unilateral sensorineural hearing loss and tinnitus. Vestibular schwannomas are common, and radiotherapy is a treatment option for symptomatic tumours [1]. Complications associated with radiotherapy include facial paralysis, hearing loss, trigeminal neuralgia, and hemifacial spasm. In addition, hydrocephalus is a potential concomitant disease [2]. Symptomatic hydrocephalus associated with vestibular schwannomas is commonly reported, particularly when cerebrospinal fluid (CSF) pathways are obstructed by a large tumour, leading to the need for ventriculoperitoneal shunt placement. Hydrocephalus may also occur without a clear CSF pathway obstruction following radiotherapy for even small schwannomas. The pathophysiology is likely due to protein release from tumour necrosis [3-5].

This case report was previously presented as a poster presentation at the British Radiosurgery Society 9th annual meeting on 24th January, 2025.

## Case presentation

**Figure 1:**
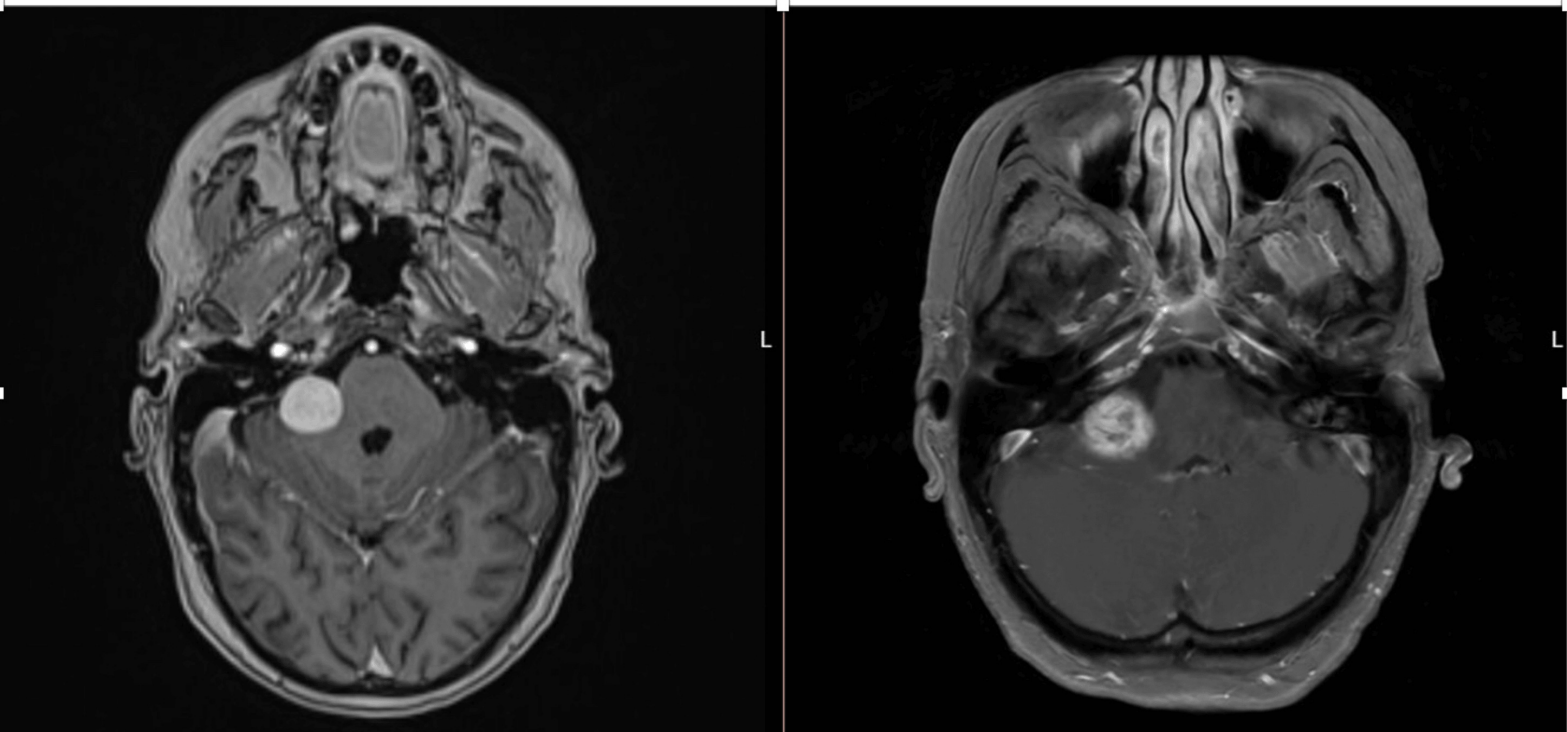
Axial T1-weighted MRI. Before radiotherapy (left), and 13 months after radiotherapy (right).

A 72-year-old woman, with no comorbidities, initially presented with some imbalance of uncertain cause. Examination at this stage showed a right sensorineural hearing loss. She underwent formal vestibular function testing, which suggested a right peripheral vestibular deficit. A subsequent MRI scan in October 2021 identified an enhancing mass in the right cerebellopontine angle and the right internal auditory meatus measuring 15 × 16 × 11 mm, which was consistent with a right vestibular schwannoma. Further surveillance MRI showed early growth at 3-4 mm of enlargement over a six-month period (Figure 1, left). Therefore, she was treated with radiotherapy 50 Gy in 30 fractions using volumetric modulated arc therapy (VMAT), which was completed in December 2022.

**Figure 2:**
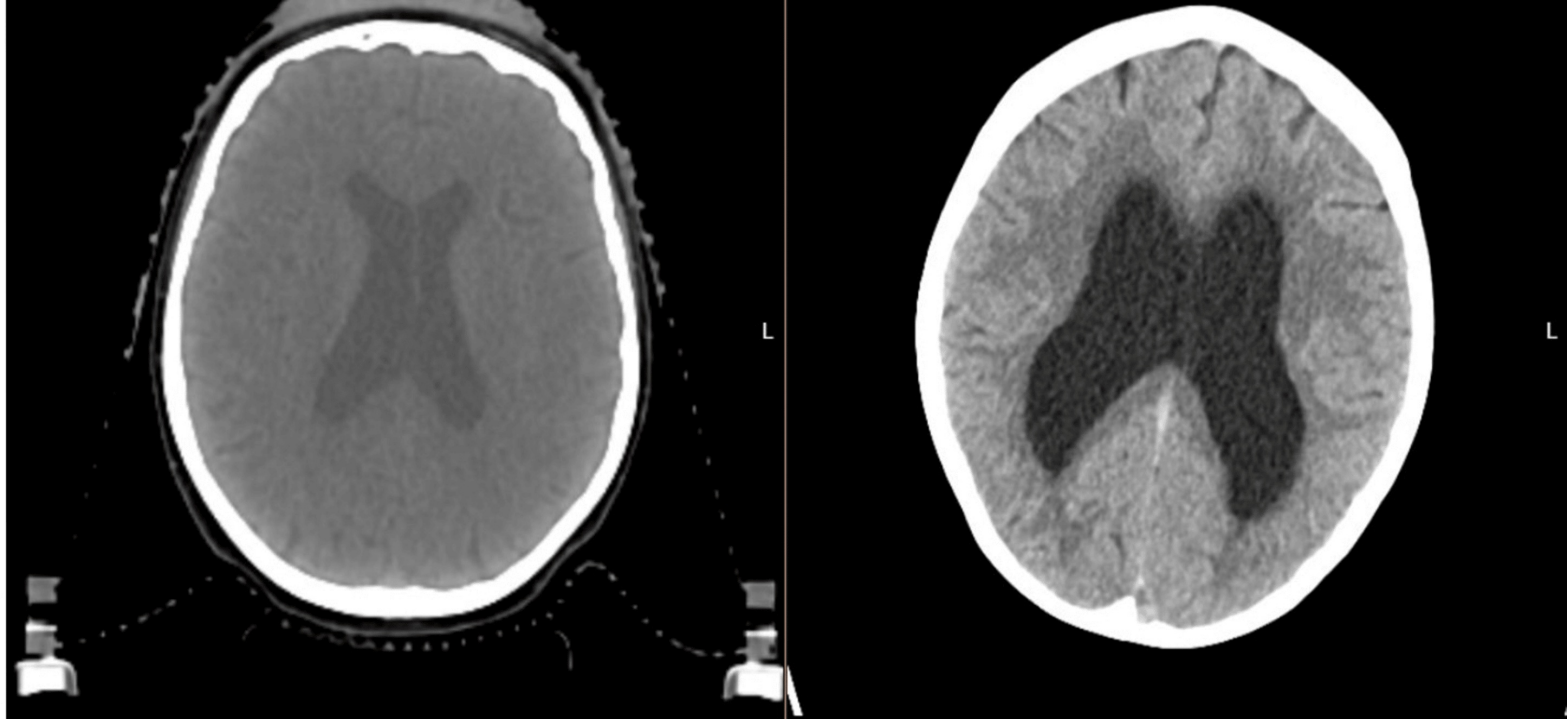
Radiotherapy planning CT before radiotherapy (left), and contrast CT 22 months after radiotherapy (right).

Sixteen months after radiotherapy, she developed intermittent slurred speech, loss of concentration, slow cognition, right facial asymmetry, and numbness. In August 2024, she was admitted to the neurosurgical team after presenting with recurrent falls and worsening of the neurological symptoms. CT of the head showed significant interval dilatation of the lateral ventricles and third ventricle (Figure 2, right). Therefore, a left ventriculoperitoneal shunt was inserted for the management of communicating hydrocephalus. MRI was not performed at that time; however, MRI in July 2024 (one month ago) showed a right acoustic schwannoma with no significant interval change in size and no interval change in the size of the ventricles.

Three weeks after the ventriculoperitoneal shunt insertion, she developed symptoms of shunt blockage, such as headache, nausea, and vomiting, with ventriculomegaly on CT scan, and shunt revision was performed. CSF results at that time showed high protein of 2.3 g/L (normal range = 0.15-0.45 g/L), red blood cell count of 107 × 10^6^/L, and white blood cell count of 4 × 10^6^/L. Subsequently, she underwent near-total resection of a growing vestibular schwannoma (29 mm), as it was important to remove the entirety of the tumour to minimise the chances of recurrent shunt blockage due to the high CSF protein content. The latest MRI scan was reassuring, and she remains under follow-up by the neurosurgical team.

## Discussion

We reported a case of communicating hydrocephalus after treatment with VMAT. Hydrocephalus is a known complication of radiotherapy for vestibular schwannomas [1]. Hydrocephalus typically develops 4-18 months post-radiotherapy, as reported in previous cases [1]. In our case, neurological symptoms appeared 16 months after radiotherapy, aligning with existing reports.

The underlying mechanisms by which hydrocephalus develops following treatment remain unclear. Three possible explanations have been proposed, namely, compression of the fourth ventricle, protein release from tumour necrosis causing obstruction of arachnoid granulations and subsequent malabsorption, and changes in CSF flow dynamics within the basilar cisterns [2-6]. Elevated protein concentration in the CSF has been associated with hydrocephalus, as multiple studies have reported intraventricular CSF protein concentrations ranging from 1.6 to 15 times higher than normal levels in cases of hydrocephalus [3,4]. Moreover, Shimizu et al. [3] reported that low concentrations of CSF proteins were associated with a reduced risk of developing hydrocephalus after radiotherapy. In our case report, CSF protein was 2.3 g/L, which was five times higher than the normal level (normal range = 0.15-0.45 g/L), which supports this hypothesis.

Another potential risk factor associated with hydrocephalus is age. Tanaka et al. [7] reported that the incidence of hydrocephalus was 12-fold higher in elderly patients (≥ 65 years of age) (25%) than in younger patients (<65 years of age) (2.1%). Moreover, it has been suggested that the increased occurrence of communicating hydrocephalus in the elderly population may be related to a decrease in the reserve capacity of CSF absorption due to ageing. In this case, the patient was over 65 years old at the time of diagnosis, which is considered a risk factor for the development of hydrocephalus.

Moreover, communicating hydrocephalus, particularly presenting with non-specific symptoms, may not be diagnosed properly and may become life-threatening. A study of over 300 vestibular schwannoma patients treated with gamma knife radiosurgery found ventricular enlargement in 20 (5.3%) cases. However, only four (1.1%) patients showed hydrocephalus symptoms at diagnosis, and all 20 had communicating hydrocephalus. The study noted that 16 of 20 patients received proactive ventriculoperitoneal shunt treatment [8]. Therefore, further research into the risk factors contributing to hydrocephalus after radiotherapy is necessary to improve early prediction and diagnosis of communicating hydrocephalus following radiotherapy for vestibular schwannomas.

## Conclusions

There have been case reports suggesting that radiotherapy for vestibular schwannomas might contribute to the development of hydrocephalus. This association is still debated, and a definitive causal relationship has not been conclusively established. One possible reason is tumour necrosis with the increased level of CSF proteins that interfere with CSF absorption at the level of the arachnoid granulations in the ventricles, which aligns with our case report. Further, other risk factors, such as age, contribute to the development of hydrocephalus according to previous studies. This evidence may be considered in planning treatment strategies for vestibular schwannoma patients who are eligible for radiotherapy.
